# Molecular discrimination of responders and nonresponders to anti-TNFalpha therapy in rheumatoid arthritis by etanercept

**DOI:** 10.1186/ar2419

**Published:** 2008-05-02

**Authors:** Dirk Koczan, Susanne Drynda, Michael Hecker, Andreas Drynda, Reinhard Guthke, Joern Kekow, Hans-Juergen Thiesen

**Affiliations:** 1 Department of Immunology, University of Rostock, Schillingallee 70, 18055 Rostock, Germany; 2 Clinic of Rheumatology, University of Magdeburg, Sophie-von-Boetticher-Straße 1, 39245 Vogelsang, Germany; 3 Leibnitz Institute for Natural Product Research and Infection Biology – Hans-Knoell-Institute e.V., Beutenbergstraße 11a, 07745 Jena, Germany

## Abstract

**Introduction:**

About 30% of rheumatoid arthritis patients fail to respond adequately to TNFα-blocking therapy. There is a medical and socioeconomic need to identify molecular markers for an early prediction of responders and nonresponders.

**Methods:**

RNA was extracted from peripheral blood mononuclear cells of 19 rheumatoid arthritis patients before the first application of the TNFα blocker etanercept as well as after 72 hours. Clinical response was assessed over 3 months using the 28-joint-count Disease Activity Score and X-ray scans. Supervised learning methods were applied to Affymetrix Human Genome U133 microarray data analysis to determine highly selective discriminatory gene pairs or triplets with prognostic relevance for the clinical outcome evinced by a decline of the 28-joint-count Disease Activity Score by 1.2.

**Results:**

Early downregulation of expression levels secondary to TNFα neutralization was associated with good clinical responses, as shown by a decline in overall disease activity 3 months after the start of treatment. Informative gene sets include genes (for example, NFKBIA, CCL4, IL8, IL1B, TNFAIP3, PDE4B, PPP1R15A and ADM) involved in different pathways and cellular processes such as TNFα signalling via NFκB, NFκB-independent signalling via cAMP, and the regulation of cellular and oxidative stress response. Pairs and triplets within these genes were found to have a high prognostic value, reflected by prediction accuracies of over 89% for seven selected gene pairs and of 95% for 10 specific gene triplets.

**Conclusion:**

Our data underline that early gene expression profiling is instrumental in identifying candidate biomarkers to predict therapeutic outcomes of anti-TNFα treatment regimes.

## Introduction

Rheumatoid arthritis (RA) is an autoimmune disease of unknown aetiology that is characterized by recruitment and activation of inflammatory cells, synovial hyperplasia, and destruction of cartilage and bone. The proinflammatory cytokine TNFα is a key mediator in the pathogenesis of RA [1]. Etanercept (Enbrel^®^; Wyeth, Cambridge, MA, USA), a soluble TNFα receptor immunoglobulin fusion protein, has been recognized as a potent biological that neutralizes TNFα [2-4]. Clinical studies on the efficacy of TNFα-blocking agents clearly show that about 30% of patients receiving this expensive therapy are nonresponders [3,5]. Although many efforts have been made to identify biomarkers for therapy response [6], no clinical or single laboratory marker exists today that allows a prediction of TNFα therapy efficacy in the individual patient. This lack of biomarker includes the newly identified specific serological marker for RA – antibodies to cyclic citrullinated peptides [7,8] – as well as genetic markers [9-12].

A number of studies have shown that the expression of individual proteins – particularly cytokines such as TNFα, IL-1β, IL-6 and IFNγ [13,14], chemokines like IL-8 and MCP1, as well as matrix metalloproteinases such as MMP1 and MMP3 [15,16] – changes during etanercept therapy. These studies were limited to a small number of genes and their corresponding proteins, and were not able to identify new markers for characterizing disease activity or to determine discriminatory markers for the prediction of therapy outcome. Van der Pouw and coworkers [17] used gene expression profiling of synovial tissue to identify subsets of RA based on molecular criteria; see also Glocker and colleagues [18].

Lequerre and colleagues described changes in gene expression signatures of mononuclear cells in RA patients 3 months after the start of treatment that were correlated with the treatment response to another TNFα inhibitor, infliximab, in combination with methotrexate [19]. They reported a significant decrease of transcript levels of eight genes regulated by TNFα-dependent pathways in nonresponders, whereas transcript levels in responders did not change significantly but were slightly increased. The effects of infliximab treatment on the long-term changes of gene expression pattern of synovial tissue and their potential to predict the outcome of infliximab-treated RA patients was investigated by Lindberg and coworkers [20]. Differentially expressed genes were involved in processes such as chemotaxis, immune function, signal transduction and inflammatory responses. The value of tissue biopsies is still under debate, and biopsies repeated in quick succession are not feasible.

The present study uses global transcriptome analysis to determine RNA expression signatures in peripheral blood cells that specify the response to anti-TNFα therapy within the first days of treatment. The objective of our approach is to discover predictive markers by analysing gene sets that are distinctly regulated in the first 3 days after anti-TNF (etanercept) administration. This short time interval was chosen to identify initially perturbed gene expression not influenced by possible changes in comedication and environmental factors occurring during longer follow-up.

We report the application of established DNA array technology (Affymetrix^®^; St. Clara, CA, USA) to monitor changes in the expression levels of mononuclear cells from peripheral blood during etanercept treatment. Among about 14,500 genes, 42 candidate genes were found suitable for use as prognostic markers for the therapeutic outcome. Using supervised learning methods, pairs and triplets derived from these genes were found to have a high prognostic value – reflected by prediction accuracies of over 89% for seven gene pairs and of 95% for 10 specific gene triplets.

## Patients and methods

### Patients

Nineteen patients (15 females, four males; mean age, 50.8 ± 11.0 years; mean duration of disease, 15.8 ± 9.4 years; all Caucasian) who met the American College of Rheumatology criteria for RA [21] were studied; for details, refer to Table 1. More than three different disease-modifying antirheumatic drugs had failed to control disease activity before etanercept was administered. The study was approved by the ethics committee of the University of Magdeburg (71/99) and all patients were asked for written consent.

**Table 1:** Patient characteristics

Patient number	Age (years)	Gender	RA duration (years)	Disease-modifying antirheumatic drugs	Steroids (mg/day)	CCP-Ab (U/ml) (*t*_0_)	DAS28		X-ray progression	Response after 3 months
									
							Baseline	3 months		
1	77	Male	21	None	5.0	644	5.45	4.69	No	Nonresponder
2	64	Male	27	Leflunomide	10.0	610	5.18	4.61	No	Nonresponder
3	43	Female	33	Methotrexate	7.5	81	4.82	0.69	No	Responder
4	65	Female	45	None	15.0	187	6.00	6.44	Yes	Nonresponder
5	63	Female	8	None	15.0	>1,600	5.83	8.37	Yes	Nonresponder
6	51	Female	17	Methotrexate	20.0	Negative	6.16	4.40	Yes	Nonresponder
7	34	Female	9	None	0.0	806	5.37	5.47	Yes	Nonresponder
8	44	Male	9	None	15.0	Negative	5.51	2.55	No	Responder
9	39	Male	1	Methotrexate	5.0	Negative	5.12	2.09	No	Responder
10	42	Female	29	Methotrexate	7.5	Negative	6.52	1.79	No	Responder
11	26	Female	2	None	0.0	Negative	4.47	1.50	No	Responder
12	48	Female	24	Leflunomide	8.0	429	5.57	2.73	No	Responder
13	47	Female	13	Cyclosporin A	10.0	96	7.11	5.29	No	Responder
14	53	Female	5	Leflunomide	8.0	1064	3.29	2.42	No	Nonresponder
15	62	Female	13	Methotrexate	0.0	Neg.	5.88	4.40	No	Responder
16	65	Female	2	Sulfasalazine/hydroxychloroquin	15.0	>1,600	7.68	5.90	No	Responder
17	42	Female	14	None	5.0	61	5.6	3.36	No	Responder
18	52	Female	8	Methotrexate	0.0	436	5.59	2.38	No	Responder
19	70	Female	14	Leflunomide	7.5	855	5.08	2.55	No	Responder

Therapeutic response was defined clinically by changes of 28-joint-count Disease Activity Score (DAS28) determined at the beginning of the study (baseline) and 3 months after the start of etanercept treatment and additionally by X-ray analysis of hands and feet after 9 to 12 months. An improvement of the DAS28 by >1.2 was considered a good response (if no progression of joint destruction were observed by X-ray analysis), a DAS28 reduction by ≤ 1.2 was considered a nonresponse. Serum antibodies to cyclic citrullinated peptide (CCP-Ab) were analysed using the Immunoscan RA ELISA CCP2 test (Euro-Diagnostica, Malmö, Sweden) according to the manufacturer's instructions (cutoff point = 25 U/ml). RA, rheumatoid arthritis.

Each patient was given a standard dose of 2 × 25 mg etanercept per week subcutaneously. Disease-modifying antirheumatic drugs and steroids remained unchanged in all patients for the first week of TNF-blocking therapy. Blood samples were taken at 7:00 a.m. before treatment (time *t*_0_; baseline), and at 72 hours after the first application of etanercept (time *t*_1_). Comedication was given after blood was taken.

Patients were assessed for overall disease activity using the 28-joint-count Disease Activity Score (DAS28) as described elsewhere [22]. Patients were categorized according to the European Leage against Rheumatism (EULAR) recommendations 3 months after the start of treatment, considering an improvement of the DAS28 >1.2 a good response. X-ray scans were read by two independent experienced physicians, but the sequence of the X-ray scans was not blinded. After reviewing X-ray scans of hands and feet, the responder group was further characterized by the absence of new bone erosions after a time interval of at least 9 to 12 months of follow up.

### Sample preparation

Peripheral blood mononuclear cells from 25 ml blood were separated on a Ficoll density gradient [23]. Using a FACSCalibur Flow Cytometer (Becton Dickinson, San Diego, CA, USA) the populations of CD3^+^, CD14^+^, CD19^+ ^and CD56^+ ^cells were determined to ensure comparability of peripheral blood mononuclear cell fractions of individual patients in the course of the study. Extraction of total RNA was performed using the Qiagen RNeasy kit (Qiagen, Hilden, Germany) including a DNA digest on-column according to the manufacturer's instructions.

### Microarray analysis

Affymetrix^® ^microarray technology (Human Genome U133A gene chip) was used to analyse the expression levels of about 18,400 transcripts interrogated by more than 22,000 probe sets. The Human Genome U95A gene chip was applied to verify array data with selected patients. Labelling and microarray processing was performed according to the manufacturer's protocol. The scanning was carried out with 3 μm resolution, 488 nm excitation and 570 nm emission wavelengths employing the GeneArray Scanner (Affymetrix, St. Clara, CA, USA). The microarray data were stored according to the MIAME standard and are available from ArrayExpress [24] (accession number E-MTAB-11).

### Quantitative real-time RT-PCR

Expression levels of a subset of genes were measured by quantitative real-time RT-PCR performed with TaqMan assay reagents according to the manufacturer's instructions on a 7900 High Throughput Sequence Detection System (Applied Biosystems, Foster City, CA, USA) using predesigned primers and probes (GAPDH Hs99999905_m1, ICAM1 Hs00164932_m1, TNFAIP3 Hs00234713_m1, IL1β Hs00174097_m1, PDE4B Hs00277080_m1, PPP1R15A Hs00169585_m1, NFKBIA Hs00153283_m1, CCL4 Hs00237011_m1, IL-8 Hs00174103_m1, ADM Hs00181605_m1).

To calculate the gene expression change of selected genes, the ΔΔC_T _method was used. According to this method, the threshold cycle values (C_T_) for specific mRNA expression in each sample were normalized to the C_T _values of GAPDH mRNA in the same sample. This provides ΔC_T _values that were used to calculate the changes of gene expression levels. Thereby, for each gene, the gene expression change in the first 3 days (ΔΔC_T_) is defined by the difference of the ΔC_T _value at day 3 (*t*_1_) and the ΔC_T _value before treatment (*t*_0_).

### Data processing and analysis

The microarray data were preprocessed using the Microarray Suite, version 5.0 (MAS5.0; Affymetrix, Santa Clara, CA, USA) in the default configuration, and were analysed by a set of algorithms.

First, an algorithm for calculation of a score *J *to rank differentially regulated genes. Basically, the *J *score introduced here is a *t *statistic, which compares the logarithm of the expression ratios *t*_1_/*t*_0 _(signal log ratios) between responders and nonresponders. Thereby, the confidence intervals of the signal log ratios provided by MAS5.0 are used. In this way, the *J *score considers interindividual differences as well as measurement errors. A higher *J *score represents a more significant differential regulation. *J *> 0 was used as the cutoff point to define genes as differentially regulated.

Second, an algorithm for learning of classifiers used for prediction of the therapy outcome on evaluation of the fold change of pairs and triplets of genes (Support-Vector Machine algorithm together with cross-validation by the leave-one-out method).

Finally, an algorithm for inference of hypothetic gene regulatory networks (modified LASSO algorithm).

These three algorithms are described in detail in Additional file 1.

Methods of multiple testing to control the type I error rates taking into account the large multiplicity (more than 22,000 probe sets) were not applied. This feature was circumvented by validating expression patterns of a selected set of genes (ICAM1, TNFAIP3, IL1B, PDE4B, PPP1R15A, NFKBIA, CCL4, IL8, ADM).

## Results

### Clinical evaluation

Before the start of treatment, all RA patients presented with a high disease activity reflected by a DAS28 (mean ± standard deviation) of 5.7 ± 0.7. Within 3 months of TNFα-blocking therapy, the disease activity decreased significantly looking at all patients as a group (DAS28 = 3.8 ± 2.1) (Table 1).

Twelve patients (patients 3, 8 to 13, and 15 to 19) were characterized by a good therapy response, as indicated by a significant reduction of the DAS28 >1.2 without progression of bone erosions as shown by X-ray scans of hands and feet. Three out of seven nonresponders (patients 4, 5 and 7) showed mild progression of bone erosion by X-ray reviewing. One patient (patient 6) was considered a nonresponder despite a good DAS28 response due to a progressive joint destruction as demonstrated by the X-ray scan. None of the clinical characteristics at baseline was significantly associated with the clinical outcome (Table 2)

**Table 2:** Comparison of clinical characteristics at baseline

Characteristic	Responder	Nonresponder	*P *value
Age (years)	48.33 (± 12.29)	58.14 (± 13.67)	0.125^a^
Gender (male)	2/12	2/7	0.603^b^
Rheumatoid arthritis duration (years)	13.5 (± 10.46)	18.86 (± 13.93)	0.353^a^
Steroids (mg/d)	6.71 (± 5.16)	10.43 (± 6.80)	0.195^a^
28-joint-count Disease Activity Score baseline	5.75 (± 0.94)	5.33 (± 0.97)	0.364^a^
Antibodies to cyclic citrullinated peptide-negative	5/12	1/7	0.333^b^
Disease-modifying antirheumatic drugs			
None	3/12	4/7	0.326^b^
Leflunomide	2/12	2/7	0.603^b^
Methotrexate	5/12	1/7	0.333^b^
Cyclosporin A	1/12	0/7	1.000^b^
Sulfasalazine/hydroxychloroquin	1/12	0/7	1.000^b^

Both patient groups show similar characteristics before therapy onset (mean ± standard deviation and number of patients, respectively). Statistical tests (^a^two-sample *t *test, ^b^exact Fisher test) were applied to check whether any of the parameters are associated with clinical outcome.

### Gene expression profiling using the U133A array

Application of Affymetrix DNA-chip technology to monitor changes in the expression profile of about 14,500 known genes in peripheral blood mononuclear cells during anti-TNFα therapy reflected a differential response by our patients as evinced by changes in the DAS28 greater than 1.2. Forty-two genes represented by 46 probe sets (Table 3) were found to be differentially regulated in therapy responders and nonresponders. The majority (40 probe sets representing 36 genes) was stronger downregulated or lesser upregulated in responders compared with nonresponders.

**Table 3:** Differentially regulated genes (probe sets) in responders and nonresponders

Symbol	Accession number	Probe set	Function	*J *value	Direction^a^	Significance^b^
Transcription/regulation of transcription						
TNFAIP3	AI738896	202643_s_at	TNFα-induced protein 3	1.1830	-	+
TNFAIP3	NM_006290	202644_s_at	TNFα-induced protein 3	0.9956	-	+
NFKBIA	AI078167	201502_s_at	NFκB enhancer in B-cell inhibitor alpha	0.4762	-	+
RUNX1	L21756	211620_x_at	Runt-related transcription factor 1	0.3940	+	+
JUN	BG491844	201464_x_at	c-jun proto-oncogene	0.1352	-	-
ZFP36L2	AI356398	201367_s_at	Zinc finger protein 36, C3H type-like 2	0.1308	-	+
SRRM2	AI655799	208610_s_at	Serine/arginine repetitive matrix 2	0.0081	+	-
ASCL1	AW950513	213768_s_at	Achaete-scute complex-like 1	0.0444	-	-
FOXO3A	AF041336	210655_s_at	Forkhead box O3A	0.0131	-	-
Immune response						
IL1B	NM_000576	205067_at	IL-1β	0.9716	-	+
IL1B	M15330	39402_	IL-1β	0.9523	-	+
CCL4	NM_002984	204103_at	Chemokine (C-C motif) ligand 4	0.8002	-	+
CCL3	NM_002983	205114_s_at	Chemokine (C-C motif) ligand 3	0.4621	-	+
CXCR4	AF348491	211919_s_at	Chemokine (C-X-C motif) receptor 4	0.2589	-	-
CXCL2	M57731	209774_x_at	Chemokine (C-X-C motif) ligand 2	0.2532	-	+
LTF	NM_002343	202018_s_at	Lactotransferrin	0.1884	-	-
PBEF1	NM_005746	217739_s_at	Pre-B-cell colony-enhancing factor 1	0.0751	-	-
IGHA1	S55735	217022_s_at	Immunoglobulin heavy constant alpha 1	0.0475	-	-
IER3	NM_003897	201631_s_at	Immediate early response 3	0.0284	-	-
Receptors, cell surface antigens, cell adhesion						
ADAM12	AU145357	215613_at	ADAM metallopeptidase domain 12 (meltrin alpha)	0.5538	-	+
ICAM1	AI608725	202637_s_at	Intercellular adhesion molecule 1 (CD54)	0.5399	-	-
SCN2B	U87555	210364_at	Sodium channel, voltage-gated, type II, beta	0.2294	+	+
Signal transduction						
PDE4B	L20966	211302_s_at	Phosphodiesterase 4B, cAMP-specific	0.4374	-	+
RAPGEF1	NM_005312	204543_at	Rap guanine nucleotide-exchange factor 1	0.2890	-	+
MYO10	AI1561354	216222_s_at	Myosin X	0.2066	-	+
PTPRD	NM_002839	205712_at	Protein tyrosine phosphatase, receptor type, D	0.1822	+	+
SOCS1	AI056051	209999_x_at	Suppressor of cytokine signaling 1	0.1239	-	-
PDE4B	NM_002600	203708_at	Phosphodiesterase 4B, cAMP-specific	0.0593	-	+
Metabolism						
LGALS13	NM_013268	220440_at	Lectin, galactose-binding, soluble, 13 (galectin 13)	0.5013	+	+
SNCA	BG260394	204466_s_at	Synuclein, alpha	0.0568	-	-
CHST3	AB017915	32094_at	Carbohydrate sulfotransferase 3	0.0366	-	+
Cellular and oxidative stress response						
CROP	AW089673	208835_s_at	Cisplatin resistance-associated overexpressed protein	0.7500	+	+
PPP1R15A	NM_014330	202014_at	Protein phosphatase 1, regulatory (inhibitor) subunit 15A	0.6886	-	+
PPP1R15A	U83981	37028_at	Protein phosphatase 1, regulatory (inhibitor) subunit 15A	0.5939	-	-
DDIT4	M_019058	202887_s_at	DNA-damage-inducible transcript 4	0.2366	-	-
SOD2	W46388	215223_s_at	Superoxide dismutase 2, mitochondrial	0.0724	-	-
ADM	NM_001124	202912_at	Adrenomedullin	0.0459	-	+
Transport						
ATP2A3	AF068220	207521_s_at	ATPase, Ca^2+ ^transporting, ubiquitous	0.227	-	-
CHRND	NM_000751	207024_at	Cholinergic receptor, nicotinic, delta	0.1977	-	+
Protein binding						
PIGO	AC004472	214990_at	Phosphatidylinositol glycan, class O	0.5216	-	+
IBRDC3	W27419	36564_at	IBR domain containing 3	0.1194	-	+
EBP49	NM_001978	204505_s_at	Erythrocyte membrane protein band 4.9 (dematin)	0.0804	-	-
FBX07	NM_012179	201178_at	F-box protein 7	0.0080	-	-
Unknown						
FSD1	NM_024333	219170_at	Fibronectin type III and SPRY domain containing 1	0.2935	-	+
HCG4P6	AF036973	215974_at	HLA complex group 4 pseudogene 6	0.1518	-	+
C20orf103	NM_013361	219463_at	Chromosome 20 open reading frame 103	0.0022	-	-

Genes were identified as differentially regulated using a modified *t*-statistic score, *J *(see Additional file 1), calculated using signal log ratios at *t*_1 _versus *t*_0 _considering 12 responders and seven nonresponders to etanercept therapy. ^a^Direction denotes genes as stronger downregulated or lesser upregulated in responders compared with nonresponders (-), and *vice versa *(+). ^b^+, significance approved by the resampling method with the modified *t *statistic on the significance level α = 0.05 (see Data processing and analysis section).

The mean of expression signals at *t*_0 _averaged over the responders (*n* = 12) and over the nonresponders (*n* = 7) did not differ significantly in these genes, with the exception of SCN2B with *P *< 0.05 (Additional file 1, Table S3a). A subset of 23 genes (represented by 27 probe sets) were approved to be differentially expressed according to the permutation test, with a significance level α = 0.05.

**Figure 1:**
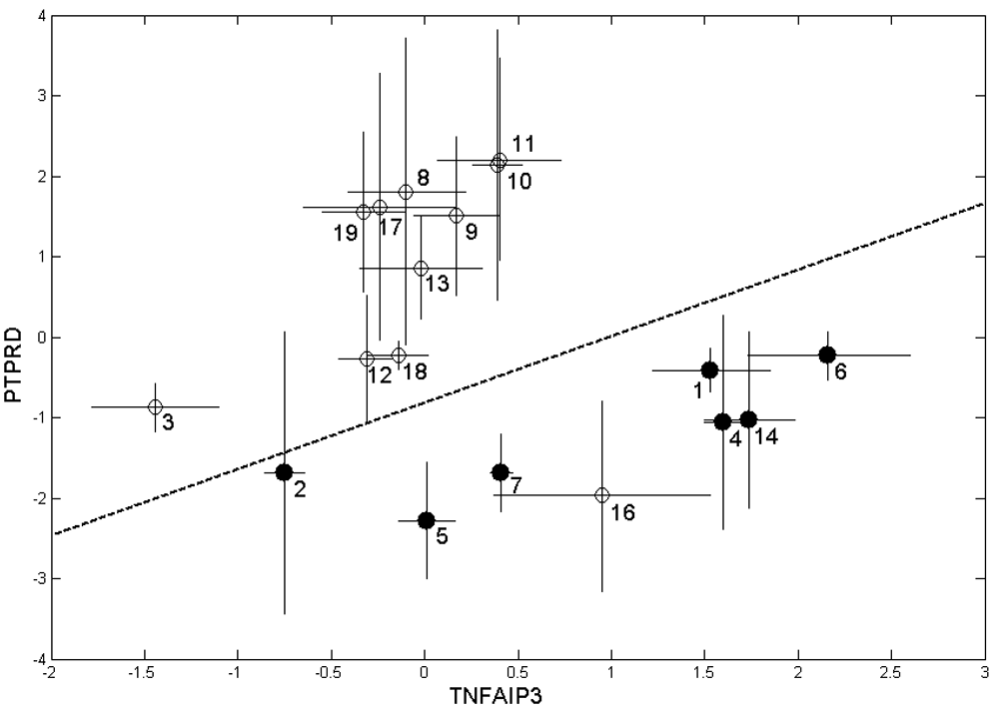
Gene expression changes of a representative predictive gene pair. Shown is the pair PTPRD [205712_at], TNFAIP3 [202643_s_at]. The pair is presented in Table 4 with a prediction accuracy of 90.5% determined using the support vector machine algorithm (signal log ratios for *t*_1 _versus *t*_0_: (○) 12 responders and (●) seven nonresponders, defined due to clinical response; bars denote the confidence intervals of the signal log ratios). Patient 16 was classified as a nonresponder based on gene expression data, but as a responder from clinical status.

All 1,035 gene pairs resulting from the 46 preselected probe sets of differentially expressed genes were examined according to their ability to clearly discriminate responders and nonresponders. For each gene pair, a set of classifiers was constructed and evaluated by cross-validation using the leave-one-out method. Seven gene pairs (Table 4) produced a prediction accuracy *Q *> 89%. Baseline levels of the selected gene pairs were not reliable in predicting the outcome as reflected by *Q*_t0log _values between 42.1% and 79.0% (Additional file 1, Table S4a). The classification performance was also insufficient when using expression levels at *t*_1 _(*Q*_t1log_). Figure 1 shows a representative example of a discriminating gene pair (*Q *= 90.5%). Only one of the 19 patients (patient 16 – preclassified to be a clinical responder) matches with the pool of nonresponders. Owing to a DAS28 score that remained reasonably high, patient 16 eventually resembles a nonresponder according to EULAR criteria.

**Table 4:** Combinations of genes predictive for the clinical outcome: gene pairs and gene triplets

Combination	Gene 1		Gene 2		Gene 3		*Q *(%)
Gene pair							
1	TNFAIP3	202643_s_at	RAPGEF1	204543_at			90.5
2	TNFAIP3	202643_s_at	PTPRD	205712_at			90.5
3	TNFAIP3	202644_s_at	PTPRD	205712_at			90.5
4	IL1B	205067_at	LGALS13	220440_at			90.5
5	CCL4	204103_at	ADAM12	215613_at			89.5
6	ADAM12	215613_at	CCL3	205114_s_at			89.5
7	FSD1	219170_at	HCG4P6	215974_at			89.5
Gene triplet							
1	CCL4	204103_at	PDE4B	211302_s_at	RAPGEF1	204543_at	99.0
2	PDE4B	211302_s_at	RAPGEF1	204543_at	CXCR4	211919_s_at	98.0
3	CCL4	204103_at	PIGO	214990_at	RAPGEF1	204543_at	96.8
4	CCL4	204103_at	FSD1	219170_at	RAPGEF1	204543_at	96.8
5	CCL4	204103_at	CCL3	205114_s_at	RAPGEF1	204543_at	96.8
6	PDE4B	211302_s_at	RUNX1	211620_x_at	RAPGEF1	204543_at	96.8
7	CCL4	204103_at	LGALS13	220440_at	RAPGEF1	204543_at	95.8
8	TNFAIP3	202643_s_at	CCL4	204103_at	RAPGEF1	204543_at	95.8
9	TNFAIP3	202643_s_at	PDE4B	211302_s_at	RAPGEF1	204543_at	95.8
10	TNFAIP3	202644_s_at	PDE4B	211302_s_at	RAPGEF1	204543_at	95.8

Gene pairs and triplets of genes with prognostic relevance for etanercept therapy in rheumatoid arthritis determined using support vector machines based on 46 selected probe sets of differentially regulated genes. Gene pairs with prediction accuracy *Q *> 89% and triplets of genes with prediction accuracy *Q *> 95% are shown. For gene function refer to Table 3.

**Figure 2:**
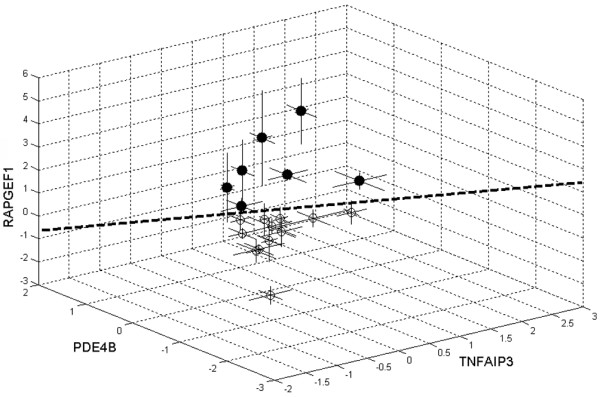
Gene expression changes of a representative predictive gene triplet. The triplet of genes TNFAIP3, PDE4B, RAPGEF1 is shown. The triplet is presented in Table 4 with a prediction accuracy of 95.8% determined using support vector machines (signal log ratios for *t*_1 _versus *t*_0_: (○) 12 responders and (●) seven nonresponders).

Finally, the separation strength of classification could be further improved by taking triplets of differentially regulated genes. Thereto, 15,180 triplets as combinations of the 46 selected probe sets were computed. Ten triplets were identified to express a prediction accuracy >95%. Figure 2 shows a three-dimensional plot of one representative triplet gene set as presented in Table 4.

### Validation of GeneChip U133A microarray data

Expression levels of a subset of genes were measured by quantitative real-time PCR for each patient and were compared with Human Genome arrays U133A and U95A (patients 1 to 11). As shown in Table 5, high correlations between the datasets obtained by three different methods of gene expression analysis were found.

**Table 5:** Validation of array data by real-time quantitative RT-PCR

Gene	Probe set		Correlation coefficient		
	
	U133A	U95A	U133A versus RT-PCR (*n* = 19)	U133A versus U95A (*n* = 11)	U95A versus RT-PCR (*n* = 11)
ICAM1	202637_s_at	32640_at	0.9329	0.8916	0.8560
TNFAIP3	202643_s_at	595_at	0.9437	0.9537	0.9792
IL1B	39402_at	39402_at	0.9443	0.9623	0.9667
PDE4B	211302_s_at	33705_at	0.8880	0.9583	0.6307
PPP1R15A	37028_at	37028_at	0.9519	0.9869	0.7649

Pearson correlation coefficients between real-time quantitative RT-PCR data (-ΔΔC_T _*t*_1 _versus *t*_0_) and the microarray data from the GeneChip U133A and U95A for five selected genes found to be differentially regulated in responders and nonresponders are presented.

In eight out of 20 genes selected for real-time quantitative RT-PCR (NFKBIA, CCL4, IL8, IL1B, PDE4B, TNFAIP3, PPP1R15A and ADM), the means of the gene expression change differed significantly for responders and nonresponders at significance level α < 0.05, as shown in Table 6. For all these genes, the means of the gene expression changes measured by quantitative real-time RT-PCR averaged over the seven nonresponders are positive, whereas those averaged over the 12 responders are negative or less positive than for the nonresponders.

**Table 6:** Gene expression analysis by real-time quantitative RT-PCR

Gene	Responder		Nonresponder		*P *value
NFKBIA	-0.227	(± 0.749)	1.053	(± 1.128)	0.008
CCL4	-0.142	(± 1.184)	1.144	(± 0.924)	0.025
IL8	-0.025	(± 1.871)	2.429	(± 2.489)	0.028
IL1B	-0.595	(± 1.680)	1.487	(± 2.191)	0.032
TNFAIP3	0.002	(± 0.895)	1.266	(± 1.510)	0.034
PDE4B	-0.276	(± 0.846)	0.534	(± 0.544)	0.037
PPP1R15A	-0.280	(± 0.935)	0.825	(± 1.225)	0.040
ADM	-0.931	(± 1.289)	0.279	(± 1.016)	0.049

Data shown are the changes of gene expression (-ΔΔC_T _*t*_1 _versus *t*_0_; mean ± standard deviation) of eight selected genes averaged over the 12 responders and seven nonresponders, and the corresponding *P *values determined by two-sample *t *test comparing the means of responders and nonresponders.

### Genetic network modelling

**Figure 3:**
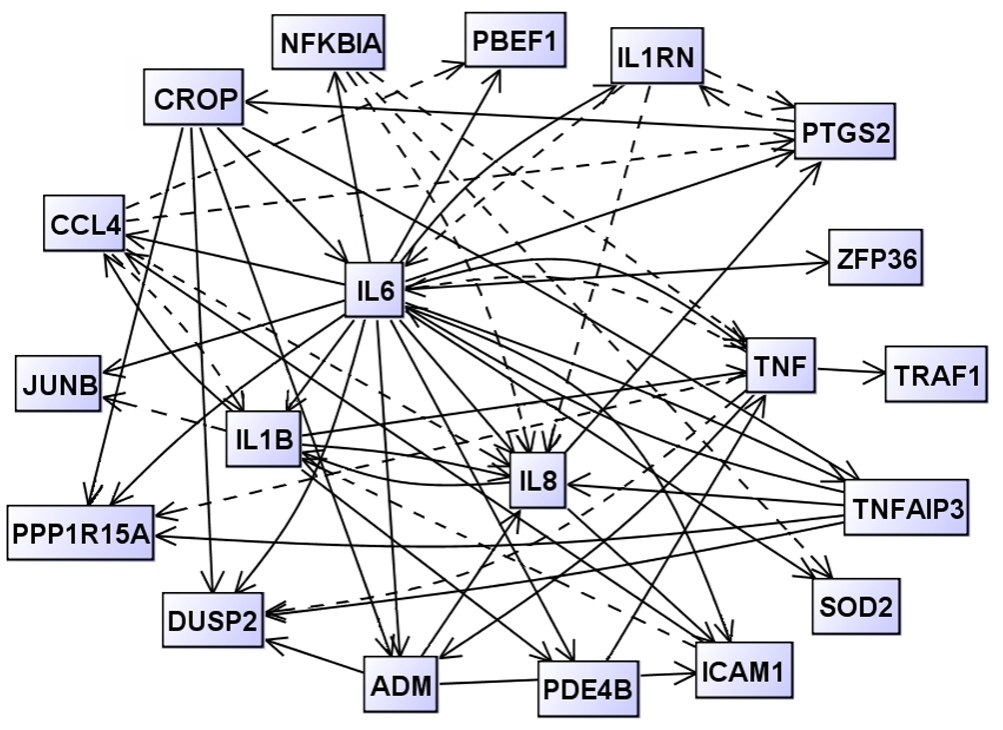
Visualization of the inferred dynamic gene regulatory network for the responder group. Each gene is represented by a node, and gene regulatory interactions are shown by directed edges. Solid lines, activating effects; dashed lines, inhibitory effects. The hypothesized network was reconstructed from quantitative real-time RT-PCR data by the modified LASSO method.

A hypothetic dynamic network was calculated (Figure 3) to reveal the underlying regulatory network that characterizes responders to the TNFα inhibitor therapy. This responder model accentuates IL-6 functions through the highest number of edges (vertex degree of 22) (see Additional file 1).

## Discussion

The goal of the present study was to identify reliable biomarkers for predicting therapy outcomes in RA patients treated with the TNFα-blocking agent etanercept. Changes of the pre-existing gene activities were monitored following the neutralization of TNFα. The Affymetrix microarray technique produced reliable semiquantitative results confirmed by comparing real-time RT-PCR results of selected genes with Affymetrix microarray results.

By applying a newly implemented criterion that takes into account the confidence intervals of the signal log ratios of gene expression [25] (see Additional file 1), 42 candidate genes (46 probe sets) were found to be differentially regulated following a single application of etanercept (Table 2). The early downregulation of expression levels secondary to TNFα neutralization includes genes involved in different pathways and cellular processes such as TNFα signalling via NFκB (TNFAIP3, NFKBIA), NFκB-independent signalling via cAMP (PDE4B), and in the regulation of cellular and oxidative stress response (PPP1R15A, DDIT4, CROP, adrenomedullin, MnSOD). The differential expression of this gene set was associated with distinct clinical responses as evinced by changes in overall disease activities 3 months after the start of treatment. The majority of the identified genes (40 probe sets) were found to be downregulated in responders compared with nonresponders. The differential expression of 27 probe sets was confirmed to be significant using a resampling method. Most importantly, changes in the expression profiles of these selected genes, particularly of pairs or triplets of genes detected 3 days after the start of treatment, were identified as being closely associated with the outcome of therapy (Additional file 1, Tables S3a, S3b). Flow cytometry analysis ruled out that changes of the expression pattern within the first 3 days of treatment were due to an altered cellular distribution of peripheral blood cells.

Two patients (patients 2 and 16) who were not predicted properly were classified as outliers by correlating clinical data and gene expression changes. Patient 2 presents a highly destructive RA, making it difficult to distinguish joint destructions in RA from destructions due to secondary osteoarthritis. Patient 16 displays the highest DAS28 score of the cohort, making it difficult to classify the patient as responder when reaching a DAS28 of 5.9, which is exceptionally high. The stratification of these two cases is hampered in their overall assessment by the limitation of tools such as the DAS28.

In contrast to changes in gene expression pattern in the first days of treatment, gene expression signatures at a single time point, here at baseline, were not reliable in predicting the clinical outcome. Diversities between RA patients on the genetic, molecular and clinical levels [17] evinced by the presence of autoantibodies (rheumatoid factor, anti-cyclic citrullinated peptide antibodies) [26] probably underline the difficulty to predict therapy outcome solely based on pretreatment expression profiles. Eventually, the differences seen in transcriptional responses to etanercept administration might either reflect the state or type of the RA disease or describe epigenomic/genomic variabilities within the patient cohort.

The reconstructed dynamic network representing responders (Figure 3) indicates that not only TNFα may play a significant role in the response to TNFα inhibitors such as etanercept. IL-6-related functionalities seem to play a key role in the responder model, while TNFα-related mechanisms are underscored in nonresponders. The functional dynamics of TNFα and IL-6 might be crucial for the outcome of an etanercept therapy. In biological terms, functionalities of anti-TNFα responses observed in nonresponding patients in comparison with responding patients might emerge due to a differential dynamic regulation of TNFα and of TNFα-dependent target gene expression, possibly also flanked by TNFα-independent mechanisms.

Responders show complex network functions of cytokines including IL-6-mediated, IL-1-mediated, and IL-8-mediated activities. Once TNFα signals are therapeutically downregulated, cytokines such as IL-6 and IL-8 become visible, possibly modulating and eventually attenuating TNF-driven inflammatory processes. This observation is in line with reports on the pleiotropic/anti-inflammatory actions of IL-6 [27], which demonstrated the role of endogenous IL-6 in controlling the levels of proinflammatory cytokines in acute inflammatory responses. The particular role of IL-6 in inflammatory conditions such as RA is presently considered in therapeutic interventions that target IL-6 or its receptor [28].

Differential changes in the expression pattern following anti-TNFα treatment can most probably be attributed to the presence of genetic heterogeneities within the group of RA patients, suggesting the presence of polymorphisms (single nucleotide polymorphisms) and/or epigenetic differences (DNA methylation patterns) in the identified genes. These polymorphisms – found in regulatory gene elements of central cytokines or downstream cascades – or the combination of single nucleotide polymorphisms as well as other types of genetic variations within these differentially regulated or associated genes, such as copy number variations, might possibly turn out to be responsible for mediating therapeutic responses as observed. This hypothesis is supported by findings that some population differences in gene expressions are attributable to allele frequency differences, in particular at regulatory polymorphisms [29].

## Conclusion

The present findings demonstrate that it is possible to predict the response of RA patients to anti-TNFα therapy at an early stage of treatment with likelihood >89% (95%) based on differentially expressed gene pairs or gene triplets. By knowing gene sets differentially regulated by TNFα-blocking therapy, additional epigenetic/genetic marker information might be obtained to circumvent the necessity of conducting cost-intensive expression studies. Along these lines, the real challenge of the listed predictory gene sets (pairs and triplets) is to validate in prospectively designed clinical trials the true accuracy and clinical value of this approach in selecting patients that profit most from a TNFα-blocking therapy.

## Abbreviations

C_T _= treshold cycle; DAS = 28-joint-count Disease Activity Score; IFN = interferon; IL = interleukin; NF = nuclear factor; PCR = polymerase chain reaction; *Q *= prediction accuracy; RA = rheumatoid arthritis; RT = reverse transcription; TNF = tumour necrosis factor.

## Competing interests

Based on these studies a patent has been applied for (PCT Patent PCT/EP03/05701, submitted 30 May 2003). The authors declare that they have no further competing interests.

## Authors' contributions

H-JT initiated and coordinated the project. JK directed the study design and the patient recruitment and clinical assessment. SD and DK played a very substantial part in the experimental work, data collection and interpretation. RG was responsible for data entry and bioinformatic analysis, assisted by MH. AD was involved in the quantitative real-time RT-PCR analysis. All authors contributed to discussions and to several drafts of the paper. All authors have seen and approved the final version.

## Supplementary Material

Additional file 1 describing in detail the microarray hybridization as well as the data processing and analysis. Click here for file
