# Supplementary material for: Molecular discrimination of responders and nonresponders to anti-TNFalpha therapy in rheumatoid arthritis by etanercept
Source: Arthritis Res Ther. 2008 May 2;10(3):R50. doi: 10.1186/ar2419 (PMC2483439; doi:10.1186/ar2419)
Supplement: Additional file 1 — describing in detail the microarray hybridization as well as the data processing and analysis. [file ar2419-S1.doc]

**Supplement**

**Microarray hybridization**

First-strand synthesis was carried out by a T7-(dT)24 primer and SuperScript II Reverse Transcriptase (Gibco BRL Life Technologies) using 10 µg total RNA. Second-strand synthesis was done according to the SuperScript Choice System (Gibco BRL Life Technologies) by E. coli DNA-Polymerase I, E. coli Ligase and RNaseH. Fragment end-polishing was performed using T4-Polymerase. An in vitro transcription reaction was used to incorporate Biotin-11-CTP and Biotin-16-UTP into the cRNA probe (BioArray HighYield RNA Transcript Labeling Kit, Enzo). The fragmented cRNA was hybridized overnight (45°C) to the Affymetrix U133A (~14.500 human gene specificities) and U95A expression arrays (~12.000 human gene specificities). Arrays were then washed using the GeneChip Fluidics Station (Affymetrix) according to the manufacturer’s protocol and stained by R-Phycoerythrin Streptavidin (Molecular Probes). This was followed by an antibody amplification procedure using a biotinylated anti-streptavidin antibody (Vector laboratories) and goat IgG (Sigma). The scanning was carried out with 3µm resolution, 488nm excitation and 570nm emission wavelengths employing the GeneArray Scanner (Hewlett Packard).

**Data processing and analysis**

Identification of differentially regulated genes

The microarray data were pre-processed by use of the Microarray Suite, version 5.0 (MAS5.0, Affymetrix, Santa Clara, CA) in the default configuration and analysed by a set of MATLAB implemented special algorithms. Since our approach is based on the assumption that the distinct therapeutic outcome is associated with a differential gene expression change in the first days after the start of therapy, we used here the signal log ratio of the microarray data at t1 versus t0. The resulting ‘signal log ratio’ (*x_pi_*) for the patient *p* with *p*=1,…, *n* (*n*=19) and the probeset (gene) *i* together with the borders of the confidence intervals ‘signal log ratio low’ (*xpi_low*) and ‘signal log ratio high’ (xpi_high) were used for the identification and ranking of differentially regulated genes using the score:

$J_{i}^{down} = \frac{\mu^{-}_{i\_low}-\mu^{+}_{i\_high}}{s_{i}\cdot\sqrt{1/n^{+}+1/n^{-}}}$ (1)

to find genes stronger down-regulated or lesser up-regulated in responders than in non-responders and

$J_{i}^{up} = \frac{\mu^{+}_{i\_low}-\mu^{-}_{i\_high}}{s_{i}\cdot\sqrt{1/n^{+}+1/n^{-}}}$ (2)

to find genes stronger up-regulated or lesser down-regulated in responders compared with non-responders. The maximum of both scores, *J_i_* = max (*J_i_^down^, J_i_^up^*), were used for identification (*J_i_* >0) and ranking of differentially regulated genes These scores are based on the t-statistic [1]. These novel scores were used to replace the commonly used fold change that corresponds the difference |*µ^-^-µ^+^*|. Our score functions use the means *µ* of its confidence interval borders ‘signal log ratios low’ and ‘signal log ratios high’ instead of the means of the ‘signal log ratios’ to take into account the measurement error. The labels plus (‘+’) and minus (‘-‘) denote the averaging over the *n^+^* responders (*n^+^* =12) and the *n^-^* non-responders (*n^-^* = 7), respectively. The standard deviation *s_i_* is calculated from the ‘signal log ratios’ by averaging over all *n* patients. According to eq. (1, 2), a gene was considered to be differentially expressed if *J_i_^down^* >0 or *J_i_^up^* >0, i.e. the averaged confidence intervals of responders and non-responders do not overlap. The significance of differential regulation were checked by re-sampling [2;3]. For re-sampling scores *J_i_^down^*(*k*) and *J_i_^up^*(*k*) according to eq. (1, 2) were calculated for a large number of permutations (*k* = 1,…, 100,000) labeled randomly by ‘+’ 12 times and by ‘-‘ 7 times. If *J_i_*(*k*), obtained from more than 95% of the randomly labeled data, is smaller than the observed score *J_i_*(o), the gene *i* is considered to be differentially expressed at the significance level α=0.05.

*Supervised learning of classifiers*

A Support Vector Machine algorithm [4;5] with linear kernel together with a cross-validation approach was applied to discover groups of genes (e.g. pairs and triplets) which are suitable for predicting the responsiveness using gene expression data. To calculate the measurement error we used for training and testing enlarged data sets, e.g. for the gene pair (*i*, *j*) and the patient *p* the data set *M_pij_* = {(*x_pi_, x_pj_*), (*x_pi_, x_pj:low_*), (*x_pi_, x_pj:high_*), (*xpi_low, x_pj_*), (*xpi_high, x_pj_*)} with *m*=5 members and for the gene triplet (*i, j, k*) the data set *M_pijk_* = {(*x_pi_, x_pj_, x_pk_*), (*xpi_low, x_pj_, x_pk_*), (*xpi_high, x_pj_, x_pk_*), (*x_pi_, xpj_low, x_pk_*), (*x_pi_, xpj_high, x_pk_*), (*x_pi_, x_pj_, xpk_low*), (*x_pi_, x_pj_, xpk_high*)} with *m*=7 members. For the cross-validation by the leave-one-out method the training was carried out *n*-times (q=1,…, n) with *n*-1 data sets {*M_pij_* or *M_pijk_* with *p*=1,…, *q* - 1, *q* + 1,…, *n*} for training and the remaining set *M_qij_ or M_qijk_* for testing. Thus, for each group, e.g. pair (*i, j*) or triplet (*i, j, k*) of genes we obtained *n*m* predictions of the responsiveness (+ or -) that may be true or false. The number *Q* as the quotient of true predictions divided by the total number *n*m* of tests was called ‘prediction accuracy’. *Q* characterizes the predictive strength of expression data of the pair (*i , j*) or triplet (*i, j, k*) of genes. The groups of genes with the largest prediction accuracy *Q* were selected to construct the classifier.

*Network reconstruction*

We used network modeling to get deeper insights into effects of the anti-TNFα therapy. By blocking TNFα, etanercept intervenes in a regulatory network of many genes mainly coding for cytokines and other immune-system-related proteins. To formulate structure and dynamics of this gene regulatory network in a mathematical model, we applied a systems biological approach called reverse engineering. The idea is to reconstruct gene regulatory interactions in the underlying system from a given amount of data.

Here the network reconstruction is based on the time-course of gene expression of a subset of differentially regulated genes, using data measured by real-time RT-PCR before start of treatment (t0), as well as after 72 (t1) and 144 hours (t2, data not shown). Such data, obtained from all patients in the study, were used to compute a dynamic linear model [6]. The interaction network inference is based on the LASSO regression [7] using the expectation maximization algorithm proposed by Grandvalet [8]. The LASSO (Least Absolute Shrinkage and Selection Operator) provides a robust estimation of a genetic network with limited connectivity and low model prediction error [9].

In short, a system of linear equations was constructed: *X*β=y*. Thereby, matrix *X* and vector *y* hold the given data in an adequate form and the vector *β* holds all unknown model parameters. Each model parameter represents the strength of regulatory control of one gene on another. Now, using LASSO regression, an estimation of the model parameters *β*^est^ can be found as described elsewhere [7-9]. The LASSO is a so-called regularization or shrinkage least squares method, i.e. its favorable properties result from a regularization term (λΣ*β*), which penalizes each parameter according to its magnitude and λ specifying the slope of the penalization.

The network inference via the LASSO was further supported by integrating prior knowledge obtained from several gene regulatory databases (e.g. TRANSPATH [10]), a text-mining tool (PathwayStudio [11]) and scientific literature. To this end, we slightly modified the modeling process by modifying the regularization term of the LASSO method. Thereby we penalized the parameters dependent on the prior knowledge: If a gene is known to up-regulate (down-regulate) the expression of another gene, the corresponding model parameter *β_i_* is not penalized if the estimation *β*^est^***_i_*** provides a positive (negative) value. This selective penalization allows a soft integration of prior biological (often uncertain) knowledge. The larger the empirically chosen λ is, the more knowledge will be integrated in the linear model. Herein, λ=0.1 was chosen. Due to the modification the proposed approach is a powerful data- and knowledge-based network inference algorithm.

Table S3a (Supplement to Table 3): Comparison of gene expression at baseline. Shown are the mean values (X) of expression signals at t0 averaged over the responders (n=12) and non-responders (n=7), as well as the standard deviations (SDX) and the results (*p*-values) of the *t*-test comparing both mean values.

| Symbol | Acc. No. | Probeset | X_t0-resp_ | X_t0-nonresp_ | SDX_t0-resp_ | SDXt0_nonresp | *p*-value |
| --- | --- | --- | --- | --- | --- | --- | --- |
| TNFAIP3  TNFAIP3  NFKBIA  RUNX1  JUN  ZFP36L2  SRRM2  ASCL1  FOXO3A  IL1B  IL1B  CCL4  CCL3  CXCR4  CXCL2  LTF  PBEF1  IGHA1  IER3  ADAM12  ICAM1  SCN2B  PDE4B  RAPGEF1  MYO10  PTPRD  SOCS1  PDE4B  LGALS13  SNCA  CHST3  CROP  PPP1R15A  PPP1R15A  DDIT4  SOD2  ADM  ATP2A3  CHRND  PIGO  IBRDC3  EBP49  FBX07  FSD1  HCG4P6  C20orf103 | AI738896  NM_006290  AI078167  L21756  BG491844  AI356398  AI655799  AW950513  AF041336  NM_000576  M15330  NM_002984  NM_002983  AF348491  M57731  NM_002343  NM_005746  S55735  NM_003897  AU145357  AI608725  U87555  L20966  NM_005312  AI1561354  NM_002839  AI056051  NM_002600  NM_013268  BG260394  AB017915  AW089673  NM_014330  U83981  M_019058  W46388  NM_001124  AF068220  NM_000751  AC004472  W27419  NM_001978  NM_012179  NM_024333  AF036973  NM_013361 | 202643_s_at  202644_s_at  201502_s_at  211620_x_at  201464_x_at  201367_s_at  208610_s_at  213768_s_at  210655_s_at  205067_at  39402_at  204103_at  205114_s_at  211919_s_at  209774_x_at  202018_s_at  217739_s_at  217022_s_at  201631_s_at  215613_at  202637_s_at  210364_at  211302_s_at  204543_at  216222_s_at  205712_at  209999_x_at  203708_at  220440_at  204466_s_at  32094_at  208835_s_at  202014_at  37028_at  202887_s_at  215223_s_at  202912_at  207521_s_at  207024_at  214990_at  36564_at  204505_s_at  201178_at  219170_at  215974_at  219463_at | 1416.8  3320.4  6543.7  59.667  728.38  656.23  1473.6  85.367  670.38  1688.9  1183.7  1711.1  767.3  5880.3  268.45  4066  1417.8  8126  1219.3    42.083  1073.6  16.858  907.53  192.18  6.7917  62.475  26.425  1259.7  32.092  4615.8  53.25  1711.5  1138.8  1394.1  1092.8  1714.5  654.63  429.02  26.358  31.508  1046.6  1162.6  4156.8  26.375  97.983  153.4 | 1230.9  3144.2  6397.9  83.929  749.17  427.81  1495.2  50.2  549.76  1088.9  621.7  970.86  330.93  6038.1  75.257  4301.4  1448.3  8695.5  1295.2  34.729  906.21  39.986  614.31  152.4  4.4429  49.743  15.643  1086  52  2942.9  30.4  1513.7  774.19  1032.9  1371.4  1563  908.86  289.49  18.729  31.729  846.39  872.67  3607.4    13.514  35.957  118.73 | 1652.1  2042.6  2316.2  50.321  506.53  356.96  449.85  87.858  562.78  3509.6  2819.8  2211.5  1958.2  1754.1  741.39  3915  1338.7  7695.4  1128.4    26.063  1076.3  9.2617  754.76  110.37  4.5135  45.139  33.372  660.64  22.968  4691.6  28.09  710.45  1640.5  1710.6  606.06  1214.9  390.9  340.76  27.189  24.214  888.69  1007.2  2513.8  16.679  76.104  65.509 | 505.35  1091  1749.1  32.466  385.73  260.12  395.04  36.613  646.47  826.5  477.86  506.06  291.92  2044.6  58.937  3697.4  856.81  4360.9  498.99  54.687  300.82  14.802  134.67  189.47  1.7999  16.206  7.7112  369.82  18.191  3570.9  22.608  354.97  352.85  287.52  720.56  511.74  1082.2  341.69  6.7056  35.078  120.82  1017.7  2095.6  5.9857  22.88  69.528 | 0.7776  0.8364  0.8874  0.2710  0.9266  0.1591  0.9175  0.3313  0.6746  0.6653  0.6119  0.4003  0.5702  0.8607  0.5057  0.8989  0.9577  0.8605  0.8693  0.6942  0.6956  0.0006  0.3282  0.5672  0.2093  0.4857  0.4167  0.5339  0.0671  0.4277  0.0852  0.5040  0.5736  0.5909  0.3792  0.7593  0.4653  0.4017  0.4803  0.9872  0.5656  0.5544  0.6328  0.0681  0.0530  0.2914 |

Table S3b (Supplement to Table 3): Comparison of gene expression changes. Shown are the mean values of log ratio R t1 versus t0 averaged over the responders (R_t1vst0-resp_) and non-responders (R_t1vst0-nonresp_), as well as the standard deviations (SDR) and the results (*p*-values) of the *t*-test comparing both mean values and the sign S of significance from Table 2.

| Symbol | Acc. No. | Probeset | R_t1vst0-resp_ | R_t1vst0-nonresp_ | D | SDR_t1vst0-resp_ | SDR_t1vst0-nonresp_ | *p*-value | S |
| --- | --- | --- | --- | --- | --- | --- | --- | --- | --- |
| TNFAIP3  TNFAIP3  NFKBIA  RUNX1  JUN  ZFP36L2  SRRM2  ASCL1  FOXO3A  IL1B  IL1B  CCL4  CCL3  CXCR4  CXCL2  LTF  PBEF1  IGHA1  IER3  ADAM12  ICAM1  SCN2B  PDE4B  RAPGEF1  MYO10  PTPRD  SOCS1  PDE4B  LGALS13  SNCA  CHST3  CROP  PPP1R15A  PPP1R15A  DDIT4  SOD2  ADM  ATP2A3  CHRND  PIGO  IBRDC3  EBP49  FBX07  FSD1  HCG4P6  C20orf103 | AI738896  NM_006290  AI078167  L21756  BG491844  AI356398  AI655799  AW950513  AF041336  NM_000576  M15330  NM_002984  NM_002983  AF348491  M57731  NM_002343  NM_005746  S55735  NM_003897  AU145357  AI608725  U87555  L20966  NM_005312  AI1561354  NM_002839  AI056051  NM_002600  NM_013268  BG260394  AB017915  AW089673  NM_014330  U83981  M_019058  W46388  NM_001124  AF068220  NM_000751  AC004472  W27419  NM_001978  NM_012179  NM_024333  AF036973  NM_013361 | 202643_s_at  202644_s_at  201502_s_at  211620_x_at  201464_x_at  201367_s_at  208610_s_at  213768_s_at  210655_s_at  205067_at  39402_at  204103_at  205114_s_at  211919_s_at  209774_x_at  202018_s_at  217739_s_at  217022_s_at  201631_s_at  215613_at  202637_s_at  210364_at  211302_s_at  204543_at  216222_s_at  205712_at  209999_x_at  203708_at  220440_at  204466_s_at  32094_at  208835_s_at  202014_at  37028_at  202887_s_at  215223_s_at  202912_at  207521_s_at  207024_at  214990_at  36564_at  204505_s_at  201178_at  219170_at  215974_at  219463_at | -0.030833  -0.016667  -0.049167  0.7175  -0.015  0.093333  0.1875  -0.57583  -0.39417  -0.36917  -0.30417  0.013333  0.1325  -0.011667  -0.3  -0.20167  -0.475  -0.51667  0.0375  -0.66167  -0.28  0.22917  -0.21917  -0.24417  0.073333  0.81083  0.0083333  -0.2275  1.0325  -0.29  -0.21167  0.14583  -0.095  -0.11833  -0.1625  -0.32333  -0.56417  0.075833  -0.2675  -0.13  -0.21  -0.30083  -0.10417  -0.51667  -0.555  -0.27583 | 0.95714  0.75  0.60571  -0.84286  0.54  0.53  -0.20714  0.73429  0.35857  1.1829  1.0986  0.99143  1.5886  0.33857  1.9571  0.68286  0.10571  0.027143  0.50571  1.2229  0.35714  -0.81714  0.52571  1.4229  1.0571  -1.1971  0.69571  0.39286  -1.28  0.17143  1.4743  -0.41286  0.87714  0.70857  0.44  0.19  0.15429  0.87714  0.57286  1.1086  0.22  0.21429  0.16857  0.5  1.4343  0.66571 | -  -  -  +  -  -  +  -  -  -  -  -  -  -  -  -  -  -  -  -  -  +  -  -  -  +  -  -  +  -  -  +  -  -  -  -  -  -  -  -  -  -  -  -  -  - | 0.57905  0.43525  0.4102  1.0592  0.5553  0.25188  0.40603  1.3344  1.5735  0.92968  0.94059  0.8161  0.55573  0.44173  1.3259  1.4796  0.98619  1.8704  0.48879  1.2205  0.67336  0.84696  0.68706  0.54545  0.38993  1.3323  0.49726  0.62009  1.3146  2.1986  1.2241  0.54333  0.9683  0.91705  0.68352  0.65959  1.0523  0.52339  0.85835  0.37192  0.64296  1.8563  1.0522  1.0219  1.6217  0.83822 | 1.0733  0.8581  0.62695  1.2591  0.89155  0.45746  0.64337  1.4581  0.47425  1.6283  1.6816  0.80489  1.6709  0.4299  1.2939  1.2694  0.98294  1.1272  0.82365  1.2817  0.67606  1.24  0.63387  1.7255  1.4446  0.73602  1.3709  0.41011  1.4873  0.52065  0.57175  0.36165  0.86404  0.86285  0.70553  0.41409  0.66733  1.7541  0.5046  1.0722  0.23123  0.22582  0.20465  0.5663  0.94689  1.0483 | 0.017528  0.018427  0.013183  0.010095  0.1104  0.014907  0.1172  0.06207  0.23891  0.016189  0.030889  0.021481  0.011994  0.11067  0.0021616  0.20435  0.23198  0.49686  0.13522  0.0053698  0.063316  0.04255  0.031618  0.0059255  0.036951  0.0019917  0.12961  0.031128  0.0025799  0.59593  0.0033827  0.027446  0.042653  0.069738  0.084474  0.082444  0.12446  0.15219  0.031272  0.0017761  0.10937  0.47984  0.51143  0.027748  0.0090679  0.045621 | +  +  +  +  -  +  -  -  -  +  +  +  +  -  +  -  -  -  -  +  -  +  +  +  +  +  -  +  +  -  +  +  +  -  -  -  +  -  +  +  +  -  -  +  +  - |

Table S4: Prediction accuracies using expression levels at baseline (t0) and after 3-day therapy (t1). The same gene pairs (A) and triplets of genes (B) that were found to be predictive for the clinical outcome of etanercept therapy based on gene expression changes in the first 3 days of therapy (listed in table 4) were used in this analysis. Here, the prediction accuracies *Q* were determined using support vector machines on the basis of logarithmized (log2) signal values at t0 (*Q_t0log_*) and t1 (*Q_t1log_*).

A

| Pair no. | Gene 1 | | Gene 2 | | *Q_t0log_*(%) | *Q_t1log_(%)* |
| --- | --- | --- | --- | --- | --- | --- |
| 1  2  3  4  5  6  7 | TNFAIP3  TNFAIP3  TNFAIP3  IL1B  CCL4  ADAM12  FSD1 | 202643_s_at  202643_s_at  202644_s_at  205067_at  204103_at  215613_at  219170_at | RAPGEF1  PTPRD  PTPRD  LGALS13  ADAM12  CCL3  HCG4P6 | 204543_at  205712_at  205712_at  220440_at  215613_at  205114_s_at  215974_at | 63.2  57.9  63.2  42.1  42.1  42.1  79.0 | 84.2  89.4  84.2  84.2  68.4  63.1  36.8 |

B

| Triplet no. | Gene 1 | | Gene 2 | | Gene 3 | | *Q_t0log_*(%) | *Q_t1log_(%)* |
| --- | --- | --- | --- | --- | --- | --- | --- | --- |
| 1  2  3  4  5  6  7  8  9  10 | CCL4  PDE4B  CCL4  CCL4  CCL4  PDE4B  CCL4  TNFAIP3  TNFAIP3  TNFAIP3 | 204103_at  211302_s_at  204103_at  204103_at  204103_at  211302_s_at  204103_at  202643_s_at  202643_s_at  202644_s_at | PDE4B  RAPGEF1  PIGO  FSD1  CCL3  RUNX1  LGALS13  CCL4  PDE4B  PDE4B | 211302_s_at  204543_at  214990_at  219170_at  205114_s_at  211620_x_at  220440_at  204103_at  211302_s_at  211302_s_at | RAPGEF1  CXCR4  RAPGEF1  RAPGEF1  RAPGEF1  RAPGEF1  RAPGEF1  RAPGEF1  RAPGEF1  RAPGEF1 | 204543_at  211919_s_at  204543_at  204543_at  204543_at  204543_at  204543_at  204543_at  204543_at  204543_at | 57.9  57.9  63.2  73.7  52.6  73.7  57.9  57.9  73.7  68.4 | 63.2  57.9  84.2  73.7  68.4  68.4  79.0  84.2  84.2  84.2 |
